# Supplementary material for: Inducible gene deletion reveals essentiality of protein kinases and a septation initiation network in Candida albicans
Source: PLoS Genet. 2026 Apr 21;22(4):e1012118. doi: 10.1371/journal.pgen.1012118 (PMC13128113; doi:10.1371/journal.pgen.1012118)
Supplement: S1 Fig — The conditional mutants (M6), which contain only the ectopically integrated gene copy, and control strains (M7), which additionally retain one of the endogenous alleles (M3 in the case of KSP1) were streaked on YPD plates and incubated for 2 days at 30°C. Both independently generated strain series are shown in each case. WT, parental wild-type strain SC5314. (PDF) [file pgen.1012118.s001.pdf]

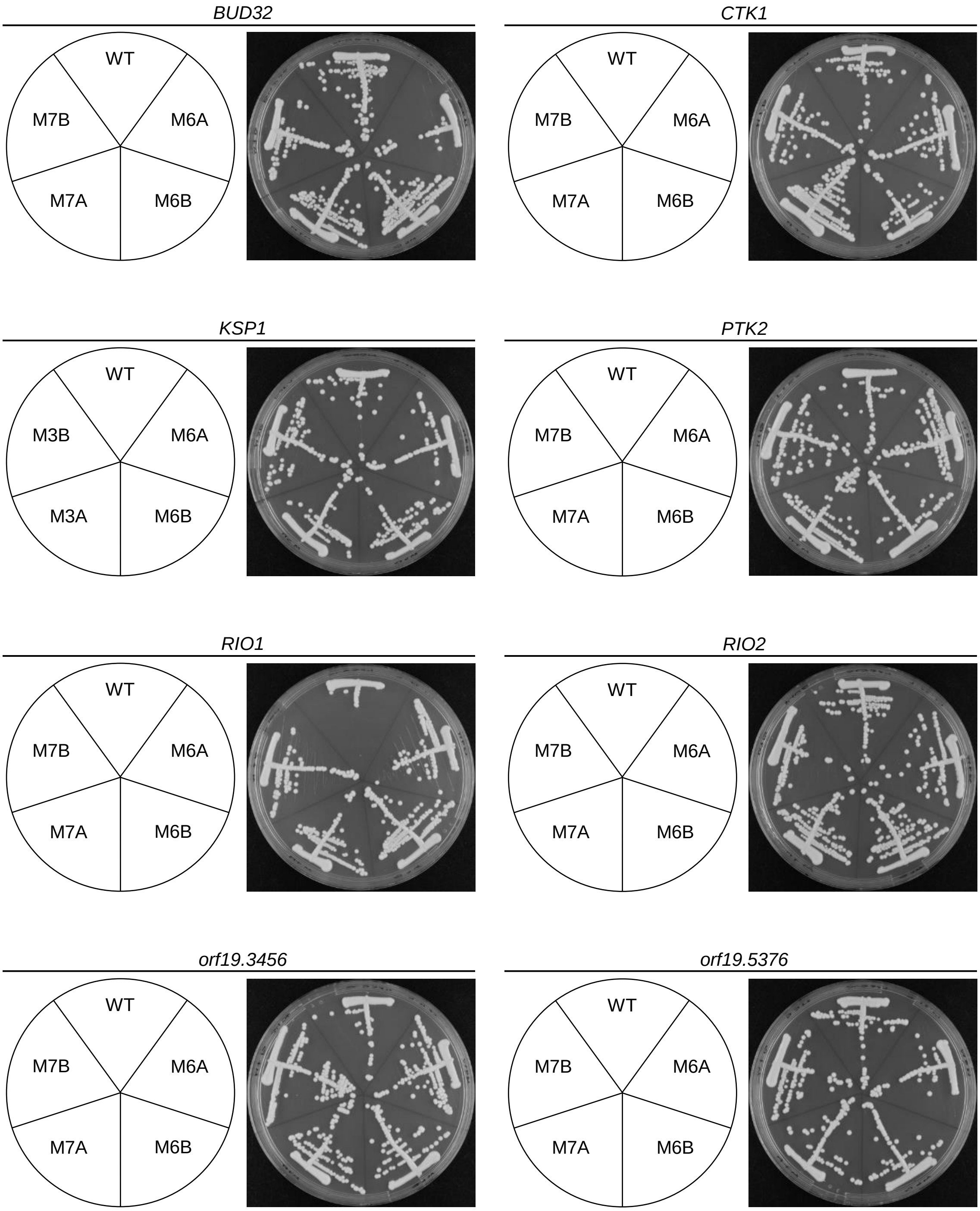

S1 Fig. Growth of the conditional mutants and control strains. The conditional mutants (M6), which contain only the ectopically integrated gene copy, and control strains (M7), which additionally retain one of the endogenous alleles (M3 in the case of *KSP1*) were streaked on YPD plates and incubated for 2 days at 30°C. Both independently generated strain series are shown in each case. WT, parental wild-type strain SC5314.
