# Supplementary material for: Inducible gene deletion reveals essentiality of protein kinases and a septation initiation network in Candida albicans
Source: PLoS Genet. 2026 Apr 21;22(4):e1012118. doi: 10.1371/journal.pgen.1012118 (PMC13128113; doi:10.1371/journal.pgen.1012118)
Supplement: S2 Fig — The M8 null mutants (M5 in the case of KSP1) and M9 control strains (M6 in the case of KSP1) were streaked on YPD plates and incubated at 30°C and at 37°C. Photographs were taken after 2, 4, and 6 days. Both independently generated strain series are shown in each case. (PDF) [file pgen.1012118.s002.pdf]

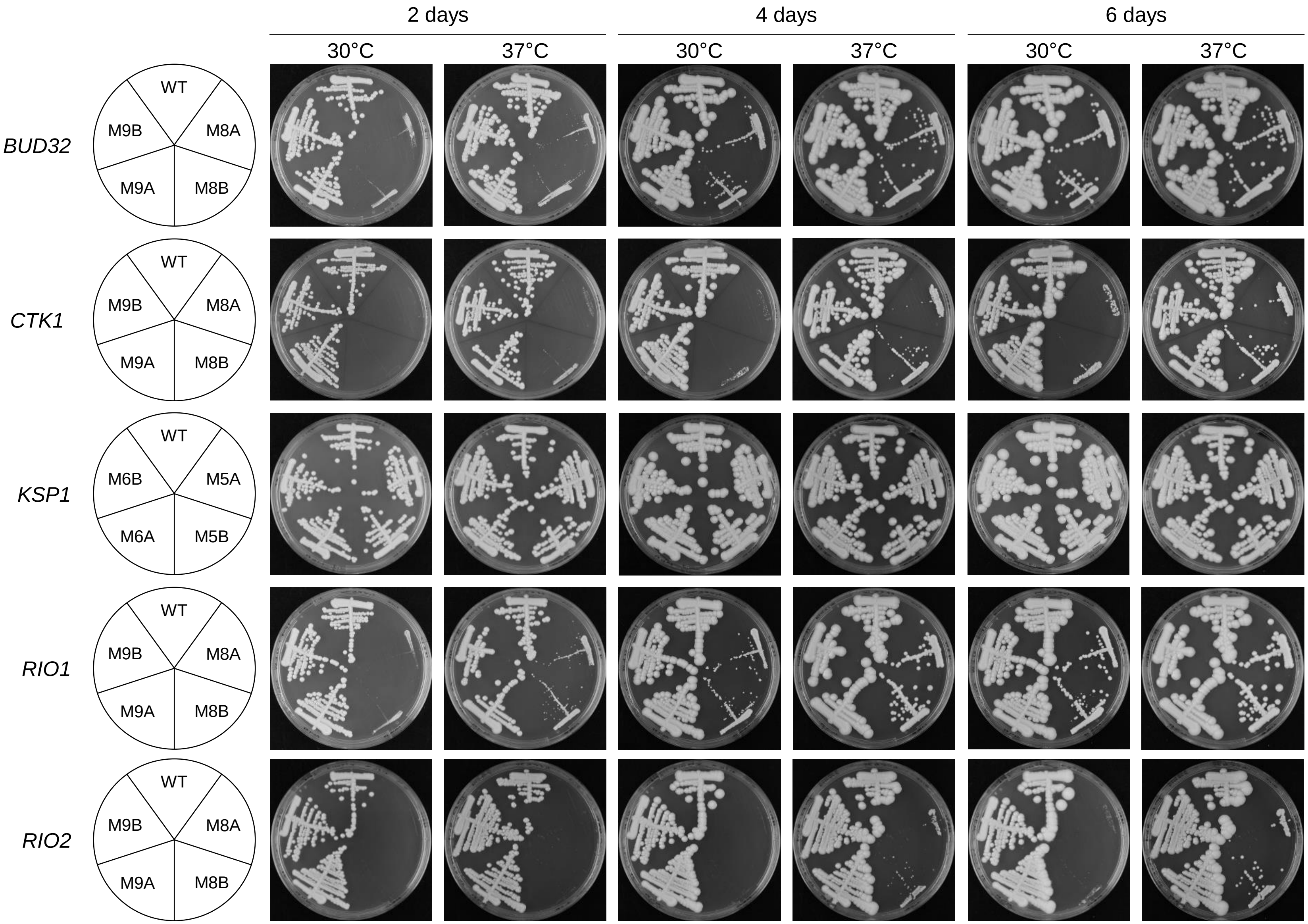

S2 Fig. Growth of viable *pek*Δ null mutants. The M8 null mutants (M5 in the case of *KSP1*) and M9 control strains (M6 in the case of *KSP1*) were streaked on YPD plates and incubated at 30°C and at 37°C. Photographs were taken after 2, 4, and 6 days. Both independently generated strain series are shown in each case.
