# Supplementary material for: Inducible gene deletion reveals essentiality of protein kinases and a septation initiation network in Candida albicans
Source: PLoS Genet. 2026 Apr 21;22(4):e1012118. doi: 10.1371/journal.pgen.1012118 (PMC13128113; doi:10.1371/journal.pgen.1012118)
Supplement: S3 Fig — (A) Four viable clones obtained after the induced gene deletion in the conditional M6 mutants (two each from strains A and B) were streaked on YPD plates and incubated for two days at 30°C. The wild-type strain SC5314 (WT) is shown for comparison. (B) Southern hybridization analysis of ClaI-digested genomic DNA of the wild-type strain SC5314, the conditional M6 mutants, and the four ptk2Δ suppressor mutants with probes from the PTK2 downstream and coding regions confirms the absence of PTK2 in the suppressor mutants. (C) Southern hybridization analysis of XhoI/SpeI-digested genomic DNA of the same strains with an ADH1 upstream probe demonstrates correct FLP-mediated excision of the ectopically integrated PTK2 copy in the suppressor mutants. A labeled size marker (M, in kb) was included in the probes. The identities of the hybridizing fragments are indicated. (PDF) [file pgen.1012118.s003.pdf]

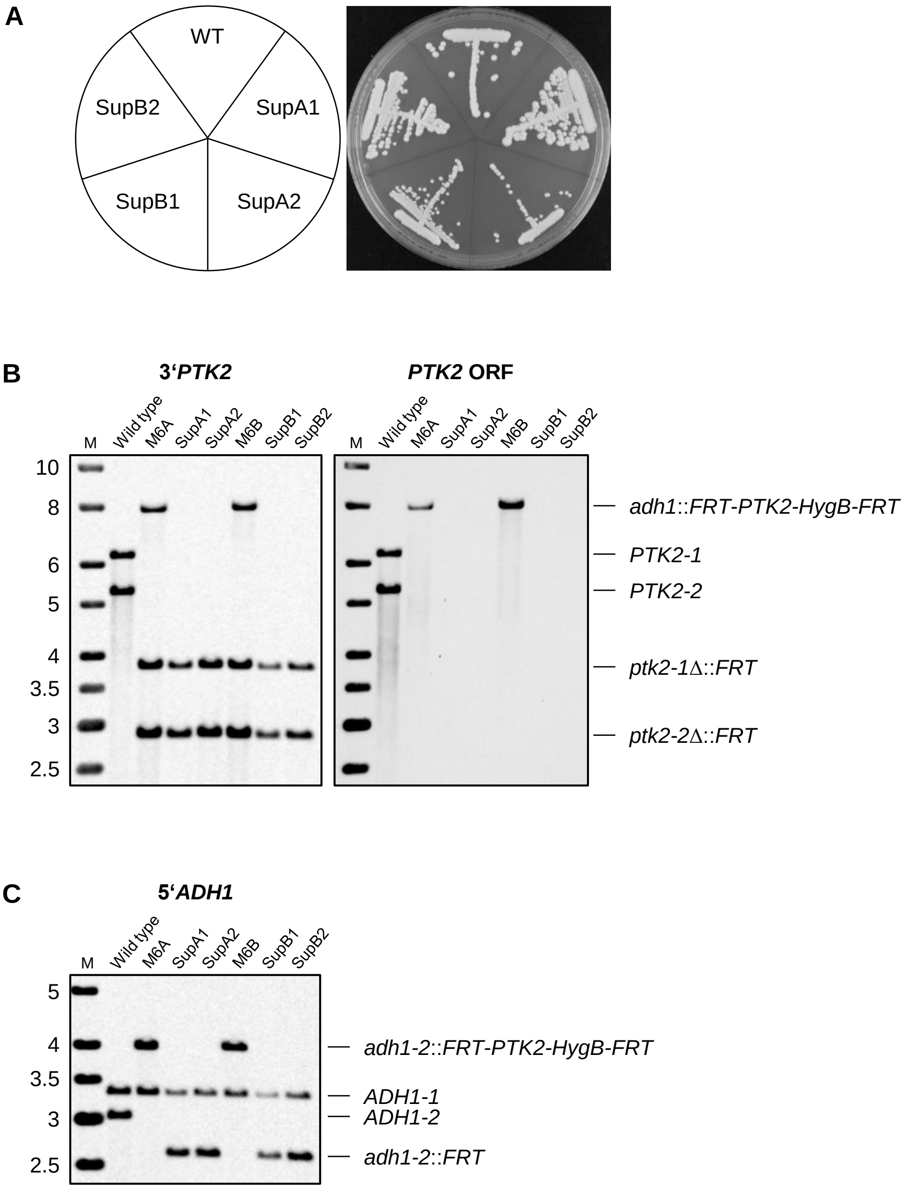

S3 Fig. Analysis of *ptk2*Δ suppressor mutants. (A) Four viable clones obtained after the induced gene deletion in the conditional M6 mutants (two each from strains A and B) were streaked on YPD plates and incubated for two days at 30°C. The wild-type strain SC5314 (WT) is shown for comparison. (B) Southern hybridization analysis of ClaI-digested genomic DNA of the wild-type strain SC5314, the conditional M6 mutants, and the four *ptk2*Δ suppressor mutants with probes from the *PTK2* downstream and coding regions confirms the absence of *PTK2* in the suppressor mutants. (C) Southern hybridization analysis of XhoI/SpeI-digested genomic DNA of the same strains with an *ADH1* upstream probe demonstrates correct FLP-mediated excision of the ectopically integrated *PTK2* copy in the suppressor mutants. A labeled size marker (M, in kb) was included in the probes. The identities of the hybridizing fragments are indicated.
