# Supplementary material for: Inducible gene deletion reveals essentiality of protein kinases and a septation initiation network in Candida albicans
Source: PLoS Genet. 2026 Apr 21;22(4):e1012118. doi: 10.1371/journal.pgen.1012118 (PMC13128113; doi:10.1371/journal.pgen.1012118)
Supplement: S4 Fig — The M7 mutants were passaged overnight in YCB-BSA-YE medium to induce FLP-mediated excison of the ectopically integrated gene copy. The cultures were diluted in water, transferred to a 35 mm culture dish, covered with YPD agar, and incubated at 30°C. Images were taken every 5 min with a DMI6000 Leica inverted microscope (S4-S5 Videos). The figure shows photographs of the cells at the indicated time points. (PDF) [file pgen.1012118.s004.pdf]

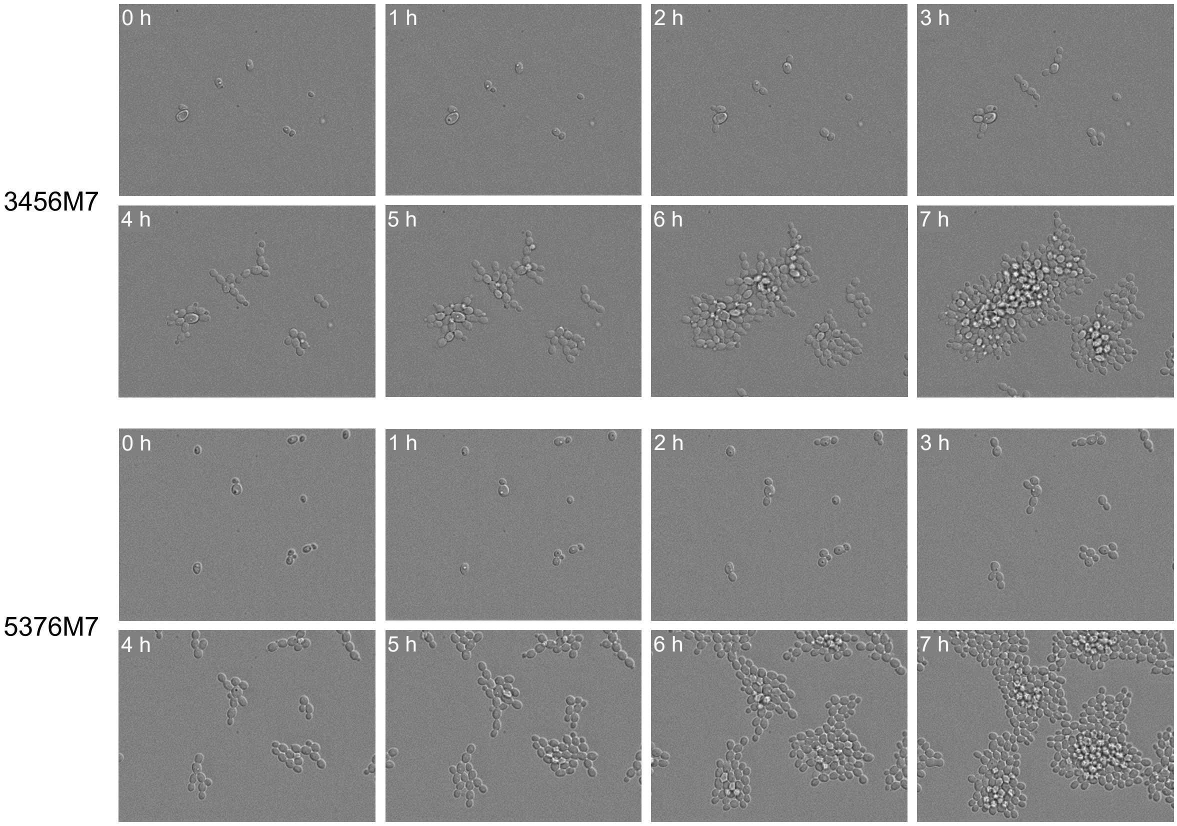

S4 Fig. Growth of *orf19.3456*Δ and *orf19.5376*Δ control strains. The M7 mutants were passaged overnight in YCB-BSA-YE medium to induce FLP-mediated excison of the ectopically integrated gene copy. The cultures were diluted in water, transferred to a 35 mm culture dish, covered with YPD agar, and incubated at 30°C. Images were taken every 5 min with a DMI6000 Leica inverted microscope (S4-S5 Videos). The figure shows photographs of the cells at the indicated time points.
