# Supplementary material for: Inducible gene deletion reveals essentiality of protein kinases and a septation initiation network in Candida albicans
Source: PLoS Genet. 2026 Apr 21;22(4):e1012118. doi: 10.1371/journal.pgen.1012118 (PMC13128113; doi:10.1371/journal.pgen.1012118)
Supplement: S5 Fig — A YPD overnight culture of the wild-type strain SC5314 was diluted 1:100 in YPD + 10% FCS and incubated for 6 h at 37°C. Aliquots of the culture were taken every 2 hours and fixed with formaldehyde. Cells were washed with PBS and stained with calcofluor white (A) or DAPI (B). Cells were imaged by DIC and fluorescence microscopy. (PDF) [file pgen.1012118.s005.pdf]

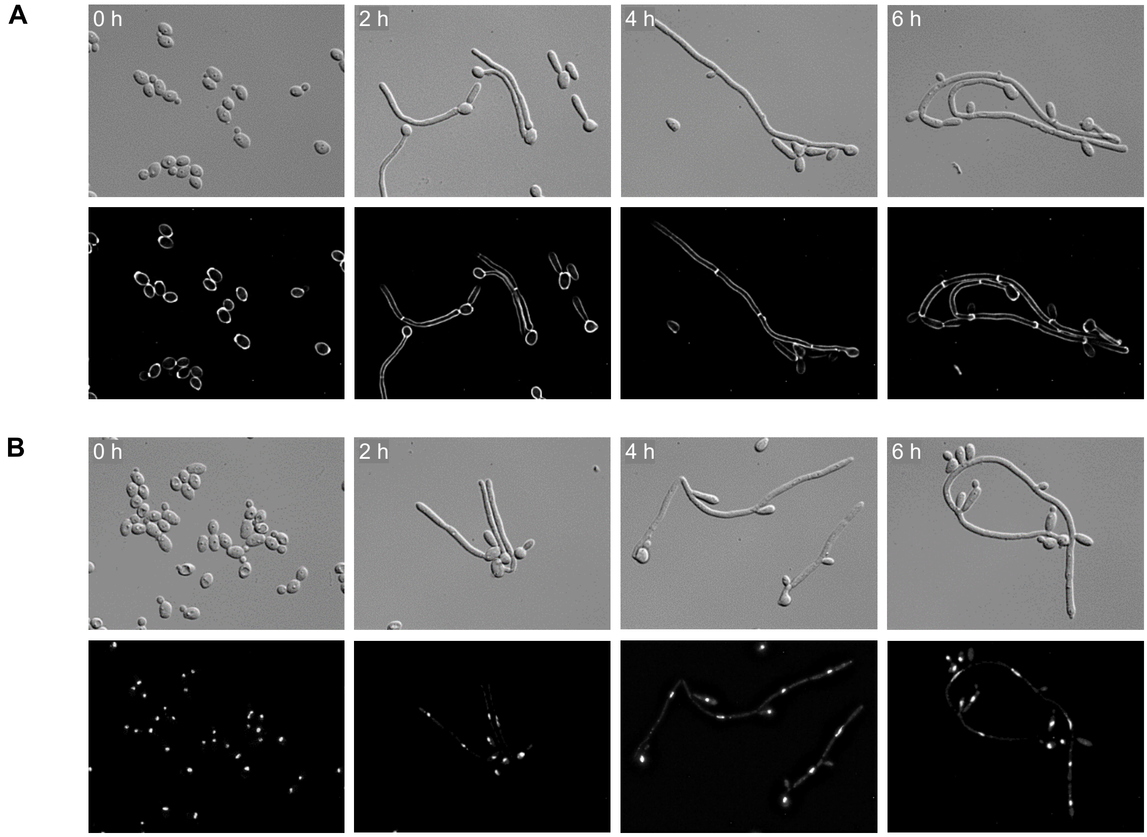

S5 Fig. Chitin and nuclei staining of serum-induced wild-type hyphae. A YPD overnight culture of the wild-type strain SC5314 was diluted 1:100 in YPD + 10% FCS and incubated for 6 h at 37°C. Aliquots of the culture were taken every 2 hours and fixed with formaldehyde. Cells were washed with PBS and stained with calcofluor white (A) or DAPI (B). Cells were imaged by DIC and fluorescence microscopy.
