# Supplementary material for: Inducible gene deletion reveals essentiality of protein kinases and a septation initiation network in Candida albicans
Source: PLoS Genet. 2026 Apr 21;22(4):e1012118. doi: 10.1371/journal.pgen.1012118 (PMC13128113; doi:10.1371/journal.pgen.1012118)
Supplement: S6 Fig — YCB-BSA-YE overnight cultures of the M7 mutants were diluted 1:100 in YPD medium and grown at 30°C. Aliquots of the cultures were taken after 4 h and fixed with formaldehyde. Cells were washed with PBS and stained with calcofluor white (A and C) or DAPI (B and D). Cells were imaged by DIC and fluorescence microscopy. (PDF) [file pgen.1012118.s006.pdf]

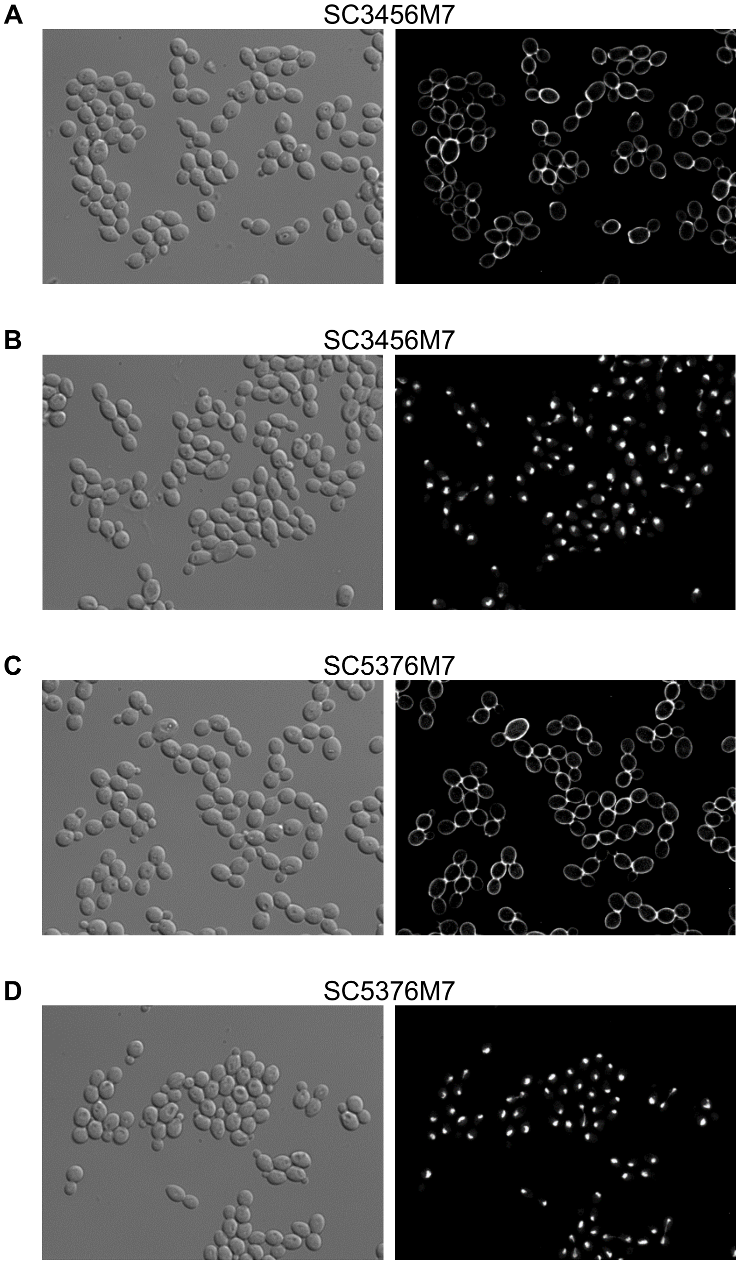

S6 Fig. Chitin and nuclei staining of *orf19.3456*Δ and *orf19.5376*Δ control strains. YCB-BSA-YE overnight cultures of the M7 mutants were diluted 1:100 in YPD medium and grown at 30°C. Aliquots of the cultures were taken after 4 h and fixed with formaldehyde. Cells were washed with PBS and stained with calcofluor white (A and C) or DAPI (B and D). Cells were imaged by DIC and fluorescence microscopy.
