# Supplementary material for: Inducible gene deletion reveals essentiality of protein kinases and a septation initiation network in Candida albicans
Source: PLoS Genet. 2026 Apr 21;22(4):e1012118. doi: 10.1371/journal.pgen.1012118 (PMC13128113; doi:10.1371/journal.pgen.1012118)
Supplement: S7 Fig — (A) Viability of strains that retain a kinase-dead allele at the endogenous locus after FLP-mediated excision of the ectopically integrated wild-type copy. YCB-BSA-YE overnight cultures of the conditional M10 mutants were appropriately diluted and the total number of cells and CFUs was determined as described in materials and methods. (B) Microscopic appearance of the kinase-dead mutants. Cells from the YCB-BSA-YE cultures were diluted, transferred to a culture dish, covered with YPD agar, and observed by video microscopy at 30°C (S6-S7 Videos). Pictures were taken at the indicated time points. (C) Kinase-dead proteins are produced at wild-type levels. Strains expressing an HA-tagged wild-type or kinase-dead allele from the endogenous genomic locus in addition to an ectopically integrated wild-type copy were grown to log-phase in YPD medium and analyzed by Western blotting with anti-HA and anti-tubulin antibodies. (D) HA-tagged kinases are functional. Strains containing a single wild-type (M2) or HA-tagged (H2) allele at the endogenous locus were grown for 2 days at 30°C on YPD plates. The wild-type strain SC5314 is shown for comparison. Results for two independently generated series of strains are shown in (A), (C), and (D). (PDF) [file pgen.1012118.s007.pdf]

A

Strain	Allele [1]	Total cells/ml	CFU/ml	CFU/total cells
SC3456M10A	*orf19.3456*^{K41R}	4.0×10^{8}	5.8×10^{5}	1.4×10^{-3}
SC3456M10B	*orf19.3456*^{K41R}	4.3×10^{8}	6.0×10^{5}	1.4×10^{-3}
SC5376M10A	*orf19.5376*^{K65R}	5.5×10^{8}	3.1×10^{5}	5.6×10^{-4}
SC5376M10B	*orf19.5376*^{K65R}	5.5×10^{8}	3.8×10^{5}	6.9×10^{-4}

[1] Allele remaining after loss of the deletable wild-type copy

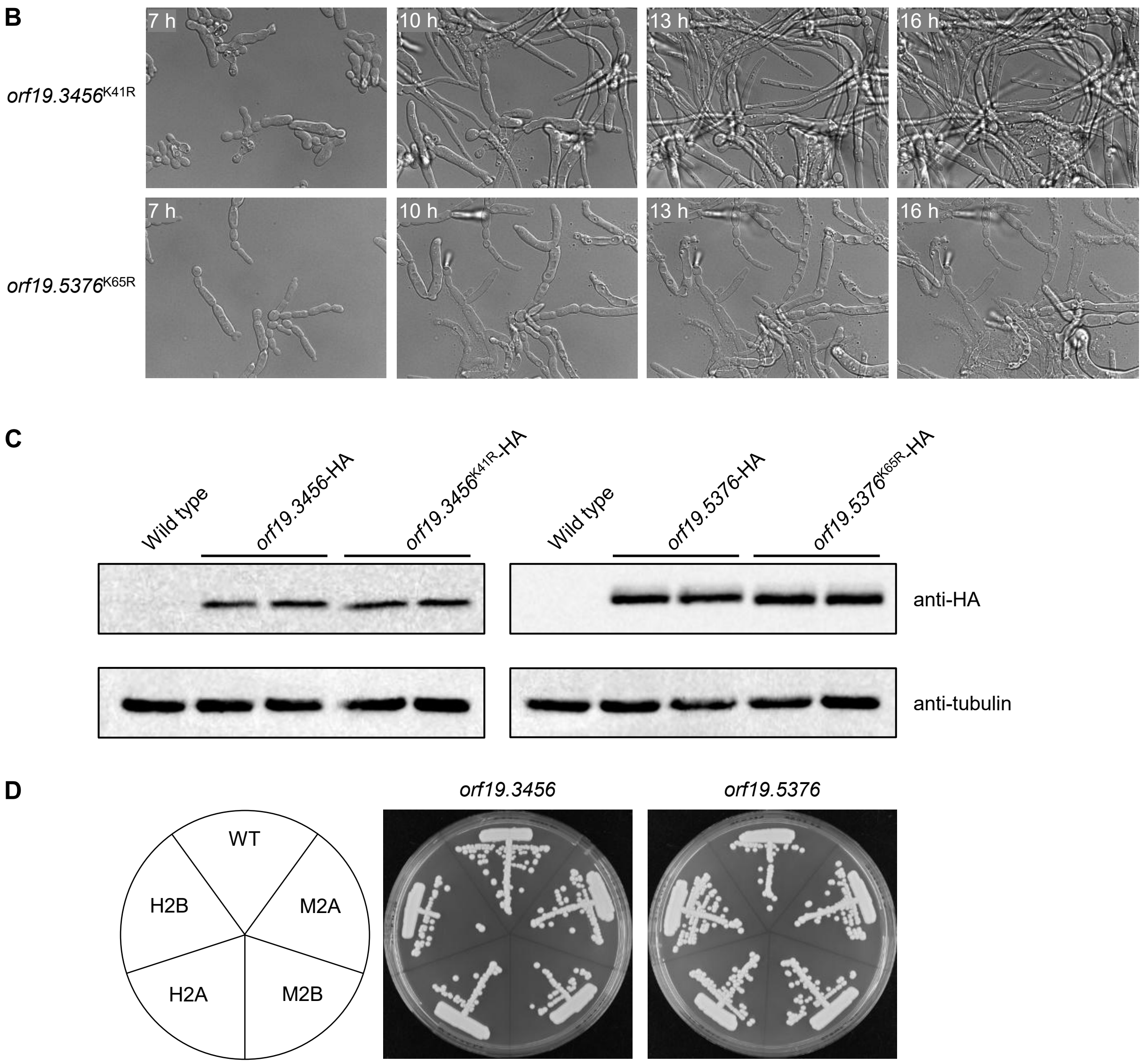

S7 Fig. Analysis of *orf19.3456* and *orf19.5376* kinase-dead mutants. (A) Viability of strains that retain a kinase-dead allele at the endogenous locus after FLP-mediated excision of the ectopically integrated wild-type copy. YCB-BSA overnight cultures of the conditional M10 mutants were appropriately diluted and the total number of cells and CFUs was determined as described in materials and methods. (B) Microscopic appearance of the kinase-dead mutants. Cells from the YCB-BSA cultures were diluted, transferred to a culture dish, covered with YPD agar, and observed by video microscopy at 30°C (S4-S5 videos). Pictures were taken at the indicated time points. (C) Kinase-dead proteins are produced at wild-type levels. Strains expressing an HA-tagged wild-type or kinase-dead allele from the endogenous genomic locus in addition to an ectopically integrated wild-type copy were grown to log-phase in YPD medium and analyzed by Western blotting with anti-HA and anti-tubulin antibodies. (D) HA-tagged kinases are functional. Strains containing a single wild-type (M2) or HA-tagged (H2) allele at the endogenous locus were grown for 2 days at 30°C on YPD plates. The wild-type strain SC5314 is shown for comparison. Results for two independently generated series of strains are shown in (A), (C), and (D).
