# Supplementary material for: Inducible gene deletion reveals essentiality of protein kinases and a septation initiation network in Candida albicans
Source: PLoS Genet. 2026 Apr 21;22(4):e1012118. doi: 10.1371/journal.pgen.1012118 (PMC13128113; doi:10.1371/journal.pgen.1012118)
Supplement: S9 Fig — A YPD overnight culture of the wild-type strain SC5314 containing a GFP-tagged CDC3 allele was diluted 1:100 in YPD + 1 µM 5-Ad-IAA and grown at 30°C. Aliquots of the culture were taken every 2 h and fixed with paraformaldehyde. Cells were washed with PBS, stained with DAPI, and imaged by DIC (left panels) and fluorescence microscopy (middle panels). The figure shows photographs of the cells at the indicated time points, including overlays of the DIC and fluorescence micrographs (right panels). (PDF) [file pgen.1012118.s009.pdf]

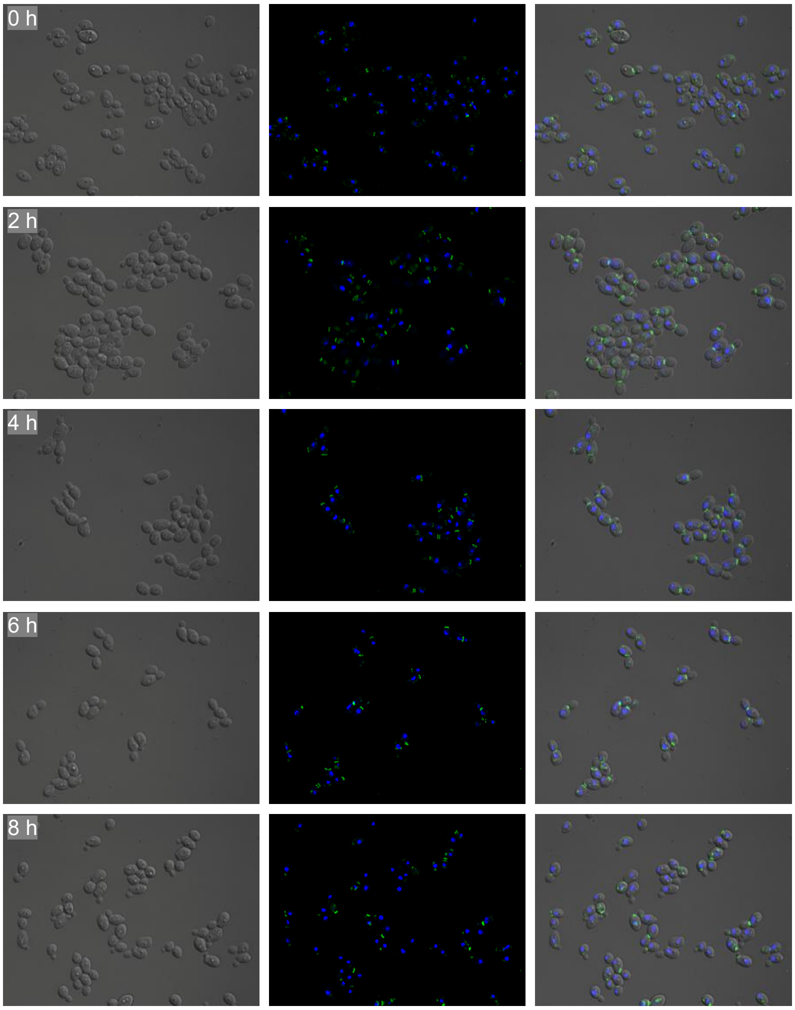

S9 Fig. Septin localization in wild-type cells. A YPD overnight culture of the wild-type strain SC5314 containing a *GFP*-tagged *CDC3* allele was diluted 1:100 in YPD + 1 µM 5-Ad-IAA and grown at 30°C. Aliquots of the culture were taken every 2 h and fixed with paraformaldehyde. Cells were washed with PBS, stained with DAPI, and imaged by DIC (left panels) and fluorescence microscopy (middle panels). The figure shows photographs of the cells at the indicated time points, including overlays of the DIC and fluorescence micrographs (right panels).
