# Supplementary material for: Inducible gene deletion reveals essentiality of protein kinases and a septation initiation network in Candida albicans
Source: PLoS Genet. 2026 Apr 21;22(4):e1012118. doi: 10.1371/journal.pgen.1012118 (PMC13128113; doi:10.1371/journal.pgen.1012118)
Supplement: S10 Fig — A YPD overnight culture of the wild-type strain SC5314 containing a GFP-tagged CDC3 allele was diluted 1:100 in YPD with 10% serum and grown at 37°C. Aliquots of the culture were taken every 2 h and fixed with paraformaldehyde. Cells were washed with PBS, stained with DAPI (A) or calcofluor white (B), and imaged by DIC and fluorescence microscopy. The figure shows photographs of the cells at the indicated time points, including overlays of the DIC and fluorescence micrographs. (PDF) [file pgen.1012118.s010.pdf]

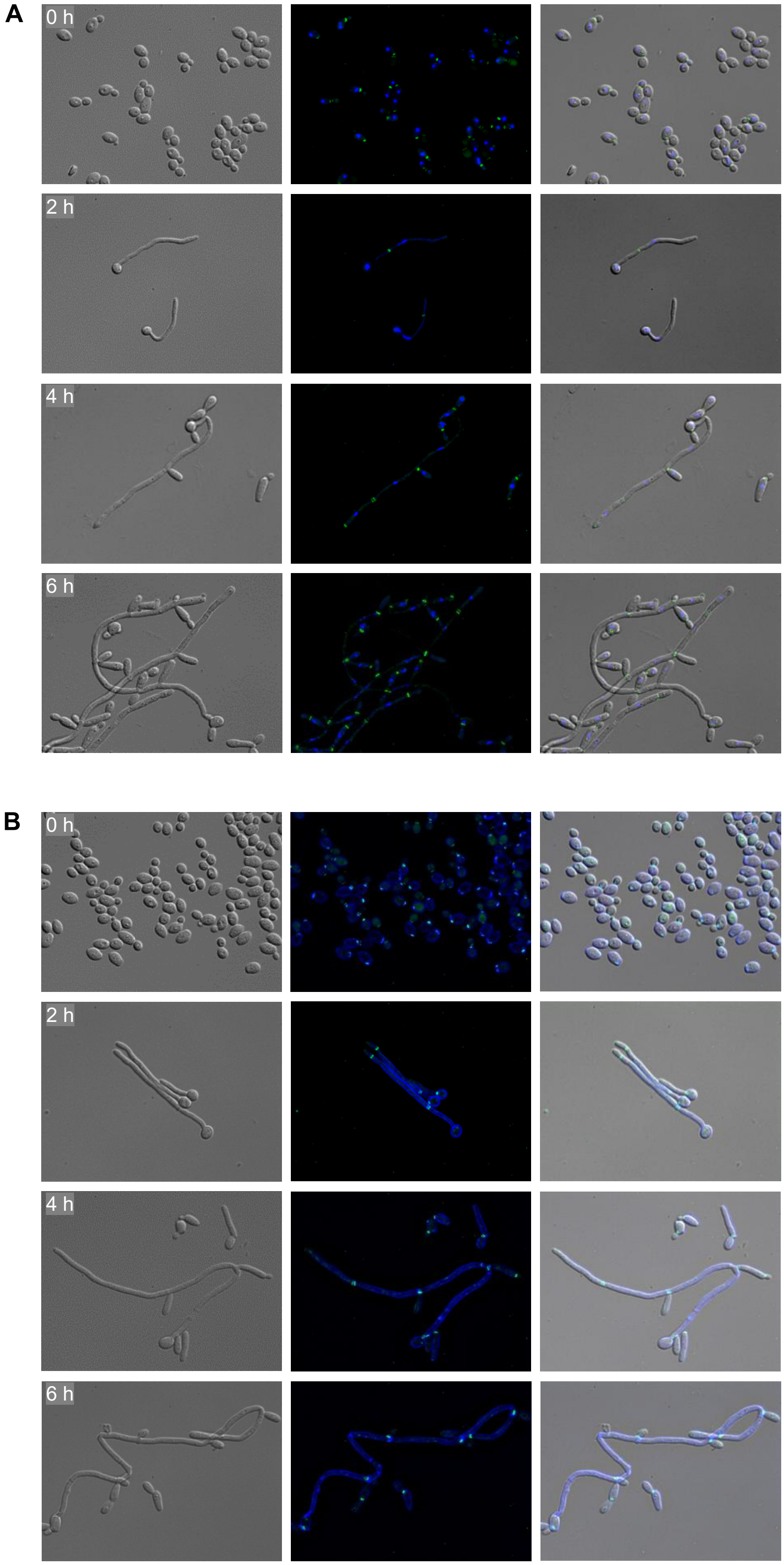

S10 Fig. Septin localization in serum-induced wild-type hyphae. A YPD overnight culture of the wild-type strain SC5314 containing a *GFP*-tagged *CDC3* allele was diluted 1:100 in YPD with 10% serum and grown at 37°C. Aliquots of the culture were taken every 2 h and fixed with paraformaldehyde. Cells were washed with PBS, stained with DAPI (A) or calcofluor white (B), and imaged by DIC and fluorescence microscopy. The figure shows photographs of the cells at the indicated time points, including overlays of the DIC and fluorescence micrographs.
