# Supplementary material for: Inducible gene deletion reveals essentiality of protein kinases and a septation initiation network in Candida albicans
Source: PLoS Genet. 2026 Apr 21;22(4):e1012118. doi: 10.1371/journal.pgen.1012118 (PMC13128113; doi:10.1371/journal.pgen.1012118)
Supplement: S11 Fig — A YPD overnight culture of the auxin-inducible orf19.3456 mutants was diluted 1:100 in YPD + 1 µM 5-Ad-IAA and grown at 30°C. Aliquots of the culture were taken every 2 h and fixed with formaldehyde. Cells were washed with PBS, stained with calcofluor white (A) or DAPI (B), and imaged by DIC and fluorescence microscopy. The figure shows photographs of the cells at the indicated time points. Identically treated control cells (the heterozygous M2 mutants containing a single untagged orf19.3456 allele) are shown in (C) and (D). (PDF) [file pgen.1012118.s011.pdf]

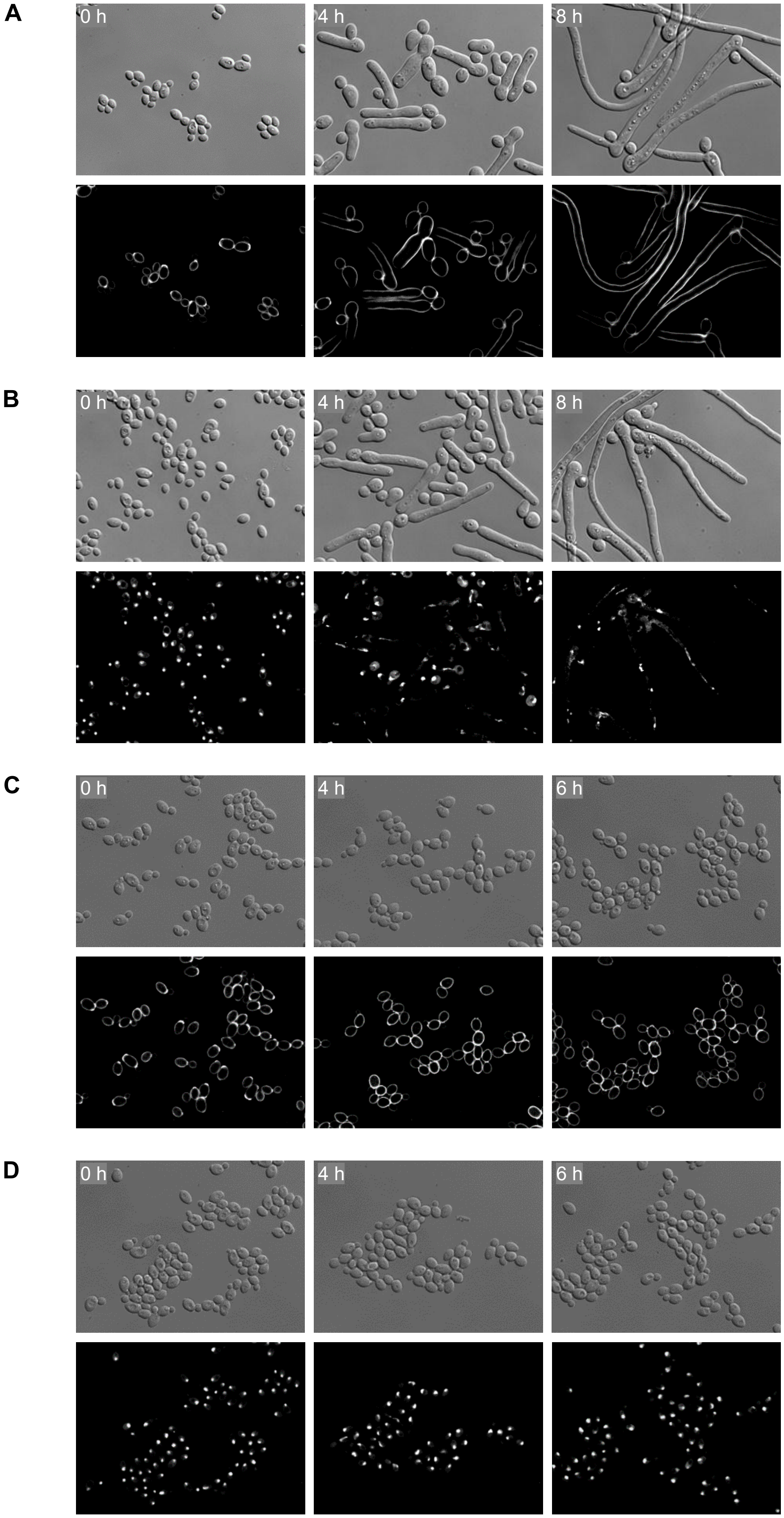

S11 Fig. Auxin-induced degradation of the *orf19.3456*-encoded kinase causes formation of aseptate, multinucleate hyphae. A YPD overnight culture of the auxin-inducible *orf19.3456* mutants was diluted 1:100 in YPD + 1 µM 5-Ad-IAA and grown at 30°C. Aliquots of the culture were taken every 2 h and fixed with formaldehyde. Cells were washed with PBS, stained with calcofluor white (A) or DAPI (B), and imaged by DIC and fluorescence microscopy. The figure shows photographs of the cells at the indicated time points. Identically treated control cells (the heterozygous M2 mutants containing a single untagged *orf19.3456* allele) are shown in (C) and (D).
