# Supplementary material for: Inducible gene deletion reveals essentiality of protein kinases and a septation initiation network in Candida albicans
Source: PLoS Genet. 2026 Apr 21;22(4):e1012118. doi: 10.1371/journal.pgen.1012118 (PMC13128113; doi:10.1371/journal.pgen.1012118)
Supplement: S13 Fig — A YPD overnight culture of the heterozygous M2 mutants containing a single untagged orf19.193 allele was diluted 1:100 in YPD + 1 µM 5-Ad-IAA and grown at 30°C. Aliquots of the culture were taken every 2 h and fixed with formaldehyde. Cells were washed with PBS, stained with calcofluor white (A) or DAPI (B), and imaged by DIC and fluorescence microscopy. The figure shows photographs of the cells at the indicated time points. (PDF) [file pgen.1012118.s013.pdf]

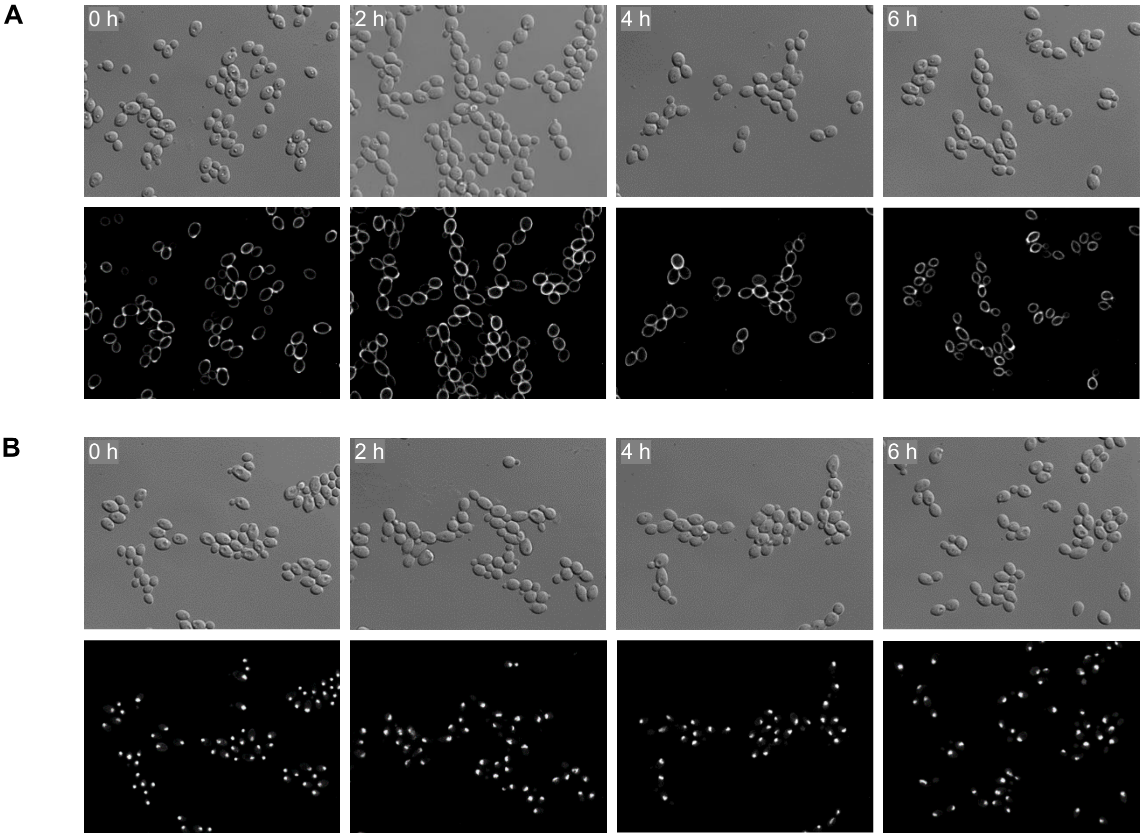

S13 Fig. Chitin and nuclei staining of auxin-treated *orf19.193* control strains. A YPD overnight culture of the heterozygous M2 mutants containing a single untagged *orf19.193* allele was diluted 1:100 in YPD + 1 µM 5-Ad-IAA and grown at 30°C. Aliquots of the culture were taken every 2 h and fixed with formaldehyde. Cells were washed with PBS, stained with calcofluor white (A) or DAPI (B), and imaged by DIC and fluorescence microscopy. The figure shows photographs of the cells at the indicated time points.
