# Supplementary material for: Inducible gene deletion reveals essentiality of protein kinases and a septation initiation network in Candida albicans
Source: PLoS Genet. 2026 Apr 21;22(4):e1012118. doi: 10.1371/journal.pgen.1012118 (PMC13128113; doi:10.1371/journal.pgen.1012118)
Supplement: Attachment — Submitted filename: Response to reviewers.docx [file pgen.1012118.s033.docx]

**Responses to the reviewers’ comments**

Our responses are highlighted in blue for clarity.

Reviewer #1:

In this study, the authors apply their inducible gene deletion strategy to definitively assign function to C. albicans protein kinase genes. They extend their analysis through time course analysis, auxin-directed protein depletion, and mass spectrometry to assign pathway relationships to several essential protein kinase genes and functionally related genes. Each new essential protein kinase gene is followed up to reach clear conclusions about signaling pathways.

This study presents a truly monumental amount of work. It is of central importance to the C. albicans community because of its implications for cell biology, virulence, regulatory networks, and drug development. It will be of interest to geneticists beyond the C. albicans community because of the clarity of thought and logic, and the elegance of the approaches.

There is an additional reason that I am especially enthusiastic about the manuscript: it presents a very high level of scholarly analysis of the literature. Essentiality and protein kinase functions have been assessed in this organism for about 25 years, so most authors seem to rely simply on the accurate but more minimal gene summaries at the CGD and other databases. I appreciated especially that the authors here really dug into the original literature to explain why results have differed over the decades.

Finally, there is a quote from the authors that I have already etched into my memory and embraced in my heart. Yeah, I may even put it on a T-shirt for all to see! It is, "The occurrence of suppressor mutations that enable (normal) growth of otherwise nonviable or poorly growing mutants is indeed an issue that must be kept in mind, especially when rare homozygous mutants are obtained using traditional gene deletion methods." In one sense it is obvious, but I cannot tell you how many times authors and experimentalists forget all about this issue as soon as they get their long sought-after mutant. I really appreciate the examples that the authors drilled down into in this study.

I have a few minor criticisms for the authors' consideration.

Line 223 "because of the immediate effect of protein depletion" I thought you might say "more immediate effect."

We agree, as even when a kinase is completely removed from a cell, its phosphorylated substrates may still be present for some time (line 228).

Lines 352-354 "...we suggest to rename these ORFs ELM1 and SID1 (and orf19.193 SRS1, for Sid1 regulatory subunit)." You might put these gene names in the abstract, because many readers may overlook them here toward the end of the paper.

This is in principle a good suggestion. However, as reviewer 2 argued that more evidence is needed to assign the gene names *ELM1* and *SID1* to *orf19.5376* and *orf19.3456*, respectively, it seems better to suggest these names only in the discussion.

Table 1. A very useful summary - thank you! (No criticism.)

Table 2. I really appreciate this summary of results, but it would be more intuitive if there were an additional column listing gene name or something like that.

We have added the gene/ORF names to the strain descriptions in Table 2.

Reviewer #2:

In this manuscript, Ramírez-Zavala et al. used an innovative inducible gene deletion strategy to assess the essentiality of eight putative essential protein kinases in the fungal pathogen Candida albicans. Of the eight kinases, PTK2, orf19.3456 and orf19.5376 were confirmed to be essential for growth under standard conditions. Under specific conditions, the authors were able to generate suppressor mutations in ptk2∆ mutants that enabled growth, namely in the Ptk2-dependent plasma membrane ATPase Pma1. Follow-up studies on the uncharacterized essential genes orf19.5376 and orf19.3456 showed the formation of filamentous cells with a defect in septum organization, which eventually lead to cell lysis. For orf19.3456, deletion mutants also exhibited multinucleated cells. To confirm the kinase activity of orf19.3456 and orf19.5376 is what was required for gene essentiality, the authors generated kinase-dead mutants, which confirmed the phenotypes of the inducible gene deletion mutants. In addition, the authors generated auxin-inducible protein depletion strains and confirmed the phenotypes of the deletion mutants. For orf19.3456, the authors performed co-IP followed by mass spectrometry to identify interacting partners, which led to the discovery of orf19.193 as a physical interactor. Depletion of degron-tagged orf19.193 resulted in the same terminal phenotype as orf19.3456 mutants, highlighting a role of this gene in C. albicans septin organization and cell cycle.

Overall, the paper presents an innovative approach to assess gene essentiality in C. albicans, emphasizing inducible gene deletion as a useful strategy to assess gene fitness in this fungal pathogen. Further, the authors establish the essentiality of several putative protein kinases that were previously uncharacterized. The authors examined the terminal phenotypes of several mutants, providing insights into the possible functions of two uncharacterized essential genes, orf19.3456 and orf19.5376. They employed complementary genetic strategies that confirmed phenotypes observed using the inducible gene deletion strategy. Nonetheless, the work could benefit from additional mechanistic insights into how orf19.3456 and orf19.5376 regulate septin organization and cytokinesis in C. albicans. To increase the impact of the manuscript, I have listed some suggestions in the point-by-point comments below:

Major comments:

1) The authors claim that orf19.3456 is the functional ortholog of S. pombe Sid1 based on cellular morphology and lack of septum formation. However, the disruption of unrelated proteins can also produce multinucleate cells and septation defects (e.g., mutants of the C. albicans RAM network). The authors should provide additional experimentation including cellular localization studies and/or functional complementation. For instance, the authors could confirm whether orf19.3456 localizes to spindle pole body in anaphase as seen with S. pombe Sid1 (Guertin et al., 2000).

We agree that determining the subcellular localization of the *orf19.3456*-encoded kinase would provide additional evidence for it being a functional ortholog of *S. pombe* Sid1. In fact, we had already tried this, but could detect neither a GFP-tagged form of the kinase by fluorescence microscopy nor the HA-tagged version by immunofluorescence microscopy. Concerning the RAM network, this is required for separation of the daughter cell from the mother cell after cytokinesis, and the phenotype of *C. albicans* mutants lacking components of the RAM network is highly different from that of our *orf19.3456* mutants (see also response to minor comment 1 below).

2) The authors performed co-IP followed by mass spectrometry to identify orf19.193 as a physical interactor of orf9.3456. While they confirmed a orf19.193 mutant showed the same phenotypes as a orf19.3456 mutant, they were unable to validate the physical interaction co-IP coupled to western blotting. To further investigate whether orf19.193 may be a regulatory subunit of orf19.3456, the authors should perform additional experimentation; for example, cellular co-localization studies to support the mass spectrometry results.

The mass spectrometry data already provided strong evidence for this particular interaction (>1,000-fold increased abundance). Nevertheless, we made a new effort to confirm the physical interaction and tagged the two proteins in a reverse fashion. The HA-tagged *orf19.193*-encoded protein could be detected by Western blotting and was specifically pulled down by the Myc-tagged *orf19.3456*-encoded kinase. These result are shown in the new Fig 9 and described in the text (lines 314-318). A BLAST search with *S. pombe* Cdc14 also identified *orf19.193* as the best hit in the *C. albicans* genome. This has now been mentioned in the revised manuscript (lines 312-314).

3) The authors showed that orf19.5376 is important for septin localization and cytokinesis, which is similar to S. cerevisiae Elm1. As discussed in Comment 1, disruption of many unrelated proteins can produce these phenotypes. Thus, additional experimental evidence to support functional orthology between orf19.5376 and S. cerevisiae Elm1 is required. For example, examining the localization of orf19.5376 to confirm it is localized to the budneck. Alternatively, the authors could attempt functional complementation studies to assess whether C. albicans orf19.5376 can complement S. cerevisiae elm1 mutants and vice versa.

We agree that demonstrating bud neck localization of the *orf19.5376*-encoded kinase would further support that it is the Elm1 ortholog in *C. albicans*. We had already tried localization studies also for this kinase, but unexpectedly could not detect GFP- or RFP-tagged forms of the kinase by fluorescence microscopy or the HA-tagged version by immunofluorescence microscopy. As suggested by the reviewer, we attempted functional complementation studies and replaced *orf19.5376* by the *ELM1* ORF (amplified from *S. cerevisiae* strain L6294). When the construct was used to transform the heterozygous M2 mutants, integration occurred only into the deleted allele and none of the transformants had replaced the remaining wild-type *orf19.5376* by the *ScELM1* ORF. We therefore integrated the construct into the deleted *orf19.5376* allele of the auxin-inducible mutants. Depletion of the AID-tagged kinase was lethal, indicating that Elm1 from *S. cerevisiae* could not substitute for its essential homolog in *C. albicans*. Nevertheless, we note that Elm1 is described in CGD as the *orf19.5376* ortholog in *S. cerevisiae*, and we provide the first experimental evidence that this is indeed the case concerning its role in septin organization (not as a Snf1-activating kinase).

4) The authors employ auxin-inducible degron-tagged kinase strains in Fig 6-Fig 9, S5 Fig, and S7 Fig. For comparison, it would be helpful to include parental controls (e.g., a strain containing single wild type allele treated with 5-Ad-IAA). For comparison of filamentous cells, it is important to include controls (e.g., serum-induced filaments), which possesses normal constrictions, septin and/or nuclei as opposed to filaments induced upon cell cycle inhibition.

We agree that the strains retaining a single copy of the wild-type gene are the formally best controls for the inducible gene deletion and auxin-inducible mutants. Since all heterozygous mutants grew normally, we used the wild-type parental strain SC5314 as control for all mutants in our subsequent assays for simplicity (see Fig 4 and Fig 5 for the inducible deletion mutants, S6 Fig for the auxin-inducible mutants in the original manuscript, now S8 Fig). As per the reviewer’s request, we have now included additional data showing that auxin-treated heterozygous *orf19.5376*∆, *orf19.3456*∆, *dbf2*∆, and *orf19.193*∆ mutants (the M2 mutants) exhibit the same typical yeast cell morphology, chitin staining, and nuclei staining as the auxin-treated parental wild-type strain SC5314 (S9 Video, S11 Video, S13 Video, S15 Video, S7C and D Fig, S10C and D Fig, S11 Fig, S12 Fig in the revised manuscript). Furthermore, we show nuclei staining and septin localization in serum-induced wild-type hyphae to exclude technical problems with staining filamentous cells (the new S9 Fig). Note that *C. albicans* hyphae do not have constrictions between cell compartments, a defining criterion for true hyphae as opposed to pseudohyphae.

5) The authors employ kinase-dead orf19.3456 and orf19.5376 strains in S4 Fig. It would be helpful for comparison to include wild type controls in both viability and microscopy assays (S4 Fig A-B).

Viability of the isogenic control strains (the M7 mutants) retaining a wild-type allele after excision of the FLP-deletable copy had already been shown in Fig 3 and quantified in Table 2. We have now included additional data (S4 Video, S5 Video, S4 Fig, S5 Fig) showing the morphology, chitin staining, and nuclei staining of these cells in addition to corresponding data for the wild-type parental strain (S1 Video, Fig 4 and Fig 5).

Minor comments:

1) The authors claim C. albicans orf19.3456 has no orthologue in S. cerevisiae (line 239-242). However, orf19.3456 appears to have 42.56% sequence identity to S. cerevisiae KIC1 (for reference orf19.3456 shows 40.23% identity to S. pombe SID1). Kic1 is a component of the well-established RAM network, which regulates polarized growth and cell separation. C. albicans RAM mutants exhibit cell separation defects and a multinucleate phenotype (Song et al. 2008), consistent with orf19.3456 mutant phenotypes. Thus, it should be addressed in the manuscript whether orf19.3456 may function as part of RAM network in C. albicans.

*KIC1* is now indeed listed in CGD as the best hit for *orf19.3456* in *S. cerevisiae* (this was not the case at the time of submission of our original manuscript). However, *C. albicans* has a clear *KIC1* ortholog, and the paper by Song *et al*. reports the phenotypes of *kic1*∆ mutants as well as deletion mutants of all other members of the RAM network (which are all viable). Contrary to our mutants, the RAM mutants all have a defect in cell separation after septum formation as well as a defect in polarized growth; they are rounder than wild-type cells and cannot form hyphae. We have verified this with our own *kic1*∆ mutants derived from the wild-type strain SC5314 (see R1 Fig, only for review purposes). This is not consistent with the phenotypes of our *orf19.3456* (and *orf19.193* and *dbf2*) mutants, which instead correspond to the reported phenotypes of other MEN/SIN pathway mutants (hyphae without septa). In our opinion there is no rationale to implicate *orf19.3456* in the RAM network.

2) The authors claim that orf19.193 belongs to the same family as S. pombe Cdc14 and thus is a putative regulatory subunit of orf19.3456 (line 301-303). However, BLASTp analysis of C. albicans orf19.193 against S. pombe Cdc14 indicates poor sequence similarity (21.71%). The authors should include supporting evidence (e.g., structural similarity analysis or conserved domains search) of why they believe orf19.193 belongs to the same family as S. pombe Cdc14.

The statement that *orf19.193* belongs to the same family as *S. pombe* Cdc14 just recapitulates protein information in CGD (Pfam entry PF08045, PANTHER entry PTHR34065), and we now have explicitly explained this in the text of the revised manuscript (lines 310-312).

3) For ease of comparison, it would be helpful to combine Fig 6C (degron-tagged kinase strain) with S6 Fig (the auxin-treated wild type control) in the same figure.

We present the data for the wild type in a separate supplementary figure for several reasons. Septin localization in *C. albicans* has been shown in numerous studies, and our data for the wild-type strain do not provide new information but only confirm that there were no technical problems in our hands. Furthermore, integrating the data from S6 Fig (now S8 Fig) into Fig 6C would require a strong reduction of the size of the micrographs, making it difficult to recognize details, or separating them from Fig 6A and B to produce another new figure, but we wish to leave the data in Fig. 6A-C together. Finally, the wild-type cells shown in S8 Fig not only are controls for the *orf19.5376* mutants but also serve as controls for the *orf19.3456* mutants shown in Fig 7C.

4) Remove the delta symbol from line 197. The header should read: “orf19.5376 encodes…”

Corrected, thanks for spotting the typing error.

Reviewer #3:

In this paper, the investigators have used a clever inducible gene deletion strategy to study a handful of kinases thought to be essential based on past inability to generate mutants in one or more reports in the human opportunistic pathogen Candida albicans. Using this strategy they found that several are actually not truly essential for growth under lab conditions, where others are. By characterizing the terminal phenotypes after gene deletion they have been able to assign a previously uncharacterized essential kinase ORF as the homolog of the S. cerevisiae ELM1 kinase involved in septin organization, bud growth and cytokinesis. For another essential uncharacterized kinase ORF, they demonstrate that it is a previously unrecognized component of the SIN pathway found in S. pombe and filamentous fungi but absent in S. cerevisiae (and thought to be generally absent in budding yeasts) and identify its putative activating subunit (also a previously uncharacterized ORF). The investigators then used a completely orthologous method, auxin-inducible protein degradation, to confirm the phenotypes and functional assignments of these two essential kinases. The application of two different methods, each producing the same phenotype, makes the study very robust and the conclusions rock solid. I find this study quite impactful as it highlights some of the problems with using permanent gene disruptions to characterize gene function and assess essentiality. In addition, the authors have provided useful new insight into the kinome of this important fungal pathogen and revealed something unexpected about a key fungal signaling pathway.

I have only a few minor comments for consideration. Otherwise, the study is well-designed and the manuscript well-written.

1. In the abstract the authors state that the product of orf19.5376 “is functionally similar” to Elm1 of S. cerevisiae. However, I think it also is the closest ortholog of Elm1 in C. albicans, correct? At least, in the Candida Genome Database Elm1 is listed as the S. cerevisiae ortholog of this kinase. I would adjust the wording to indicate this.

CGD indeed lists *ELM1* as the ortholog of *C. albicans* *orf19.5376* in *S. cerevisiae*, although a BLAST search for homologs of the *orf19.5376*-encoded kinase in SGD yields higher scores for Sak1 and its paralog Tos3 than for Elm1 (a reverse BLAST search with ScElm1 indeed yields *orf19.5376* as the top hit, as stated in the text, lines 204-205). Unlike *ELM1* in *S. cerevisiae*, *orf19.5376* has no detectable role in Snf1 activation in *C. albicans*. Therefore, we report in the abstract only our own experimental data showing that *orf19.5376* has a role in septin organization and in that respect is similar to *ELM1* of *S. cerevisiae*, especially as reviewer 2 thinks that more experimental data are needed to prove that *orf19.5376* is the functional Elm1 ortholog (see major comment 3 by reviewer 2).

2. Also in the abstract I would go ahead and state the name of the S. pombe ortholog in the SIN pathway at the end (rather than just calling it the orf19.3456-encoded kinase).

Again, due to the reservations expressed by reviewer 2, it seems better to suggest a gene name for *orf19.3456* only in the discussion. As we state in the abstract, the SIN is known also from filamentous fungi such as *Aspergillus*, in which the Sid1 ortholog is termed SepL.

3. One concern with the conclusion about the hyperpolarized “hyphal”-like structures being aseptate is that there is no control showing septa in wild-type hyphae. I understand the structures that form in these induced mutants are probably not true hyphae or pseudohyphae and that septa can be seen between some of the yeast-form cells, but it would be nice to know that the imaging method could reveal septa in the hyphal-like structures if they were present. This could be accomplished simply by inducing hyphae in the wild-type parent strain and showing the septa for comparison under identical imaging and processing conditions.

This has also been suggested by reviewer 2 (major comment 4), and we have included a figure showing septa, nuclei, and septin localization in wild-type hyphae (S9 Fig).

4. The authors conclude that the mutants exhibit cytokinesis defects (e.g. lines 179-180). While this may be true, cytokinesis has not been explicitly monitored. I feel this conclusion would require assessing formation and contraction of the actomyosin ring because there are many mutants in other pathways that give rise to a similar hyperpolarized cell phenotype in this organism. The results are consistent with this possibility but maybe the conclusion is worded too strong. Or the authors can provide more explanation for why they conclude cytokinesis is defective – its possible I am missing something.

We conclude that both *orf19.5376* and *orf19.3456* mutants have a cytokinesis defect because the pseudohyphae and hyphae, respectively, produced by these mutants do not have (normal) septa, which would become visible after contraction of the actomyosin ring, completing cytokinesis. We also did not detect the septin double ring, which allows formation of the actomyosin ring, in the mutants, so we do not expect that the construction of new reporter strains for visualizing the actomyosin ring would help. Nevertheless, we have toned down the sentence and replaced “demonstrate” by “indicate” (lines 183-185).
