# Supplementary material for: Inducible gene deletion reveals essentiality of protein kinases and a septation initiation network in Candida albicans
Source: PLoS Genet. 2026 Apr 21;22(4):e1012118. doi: 10.1371/journal.pgen.1012118 (PMC13128113; doi:10.1371/journal.pgen.1012118)
Supplement: Attachment — Submitted filename: Response_to_reviewers_auresp_2.docx [file pgen.1012118.s034.docx]

**Response to the reviewer’s comment**

Reviewer #3: I still think this is a great study and the overall rigor is high and suitable for publishing in PLoS Genetics. I only had a few comments on the initial submission and most were addressed adequately. Unfortunately, one of my concerns was not addressed as I had intended, possibly due to a lack of detail on my part. I had suggested that wild-type hyphae be shown with calcofluor white staining side-by-side with the two kinase mutants to show what normal septa would look like and validate the conclusion that the mutants lack septa in their hyperpolarized structures. The authors added new Figure S9 to address this, but unfortunately it includes Cdc3-GFP signal demarking the septin rings, which overlaps with and occludes the calcofluor white signal at the septa. This figure also does not use the same imaging and image processing method as the figures I was referring to - Figure 5A and Figure 8C. I still think it is important that wild-type hyphal controls (just SC5314) be included specifically for these figures, highlighting normal septum signal with this imaging procedure.

Response: Since the previous reviewer 2 had a similar comment but also asked us to show septin localization in serum-induced wild-type hyphae, we had performed this control experiment with wild-type cells expressing a *GFP*-tagged *CDC3* allele. We have now included an additional supplementary figure (S5) showing serum-induced hyphae of the untagged wild-type strain SC5314 that were processed for microscopy in the same way as the *orf19.3456*Δ and *orf19.5376*Δ mutants shown in Fig 5 and the auxin-induced mutants in other figures. While the reviewer requested only visualization of septa by chitin staining, we also include staining of nuclei with DAPI in the new S5 Fig to match the data shown for the mutants in Fig 5 (otherwise readers might wonder why we show only chitin staining for wild-type hyphae).
